# α-Mangostin Alleviated Inflammation in Rats With Adjuvant-Induced Arthritis by Disrupting Adipocytes-Mediated Metabolism-Immune Feedback

**DOI:** 10.3389/fphar.2021.692806

**Published:** 2021-07-07

**Authors:** Ying-Hao Hu, Jun Han, Lin Wang, Chao Shi, Yan Li, Opeyemi Joshua Olatunji, Xiu Wang, Jian Zuo

**Affiliations:** ^1^ Department of Traditional Chinese Medicine, the First Affiliated Hospital of Wannan Medical College (Yijishan Hospital), Wuhu, China; ^2^ Research Center of Integration of Traditional Chinese and Western Medicine, Wannan Medical College, Wuhu, China; ^3^ Drug Research and Development Center, School of Pharmacy, Wannan Medical College, Wuhu, China; ^4^ Department of Pharmacy, the First Affiliated Hospital of Wannan Medical College (Yijishan Hospital), Wuhu, China; ^5^ Faculty of Traditional Thai Medicine, Prince of Songkla University, Hat Yai, Thailand; ^6^ Key Laboratory of Non-coding RNA Transformation Research of Anhui Higher Education Institution, Wannan Medical College, Wuhu, China

**Keywords:** macrophage, rheumatoid arthritis, PPAR-γ, adjuvant- induced arthritis, fat metabolism, adipocytes

## Abstract

A previously identified anti-rheumatic compound *α*-mangostin (MAN) possesses notable metabolism regulatory properties. In this study, we investigated the immune implication of MAN-altered fat metabolism on adjuvant-induced arthritis (AIA) in rats. Seven days after AIA induction, the rats received oral treatment of MAN at 50 mg/kg/day for 30 days. Metabolic indicators and basic clinical parameters were evaluated using samples collected on day 20 and 38 since immunization. Expression of nicotinamide phosphoribosyltransferase (NAMPT), sirtuin 1 (SIRT1), peroxisome proliferator activated receptor gamma (PPAR-*γ*), stearoyl-coa desaturase 1 (SCD-1), toll like receptor 4 (TLR4), prostaglandin-endoperoxide synthase 2 (COX-2), (*p*)-JNK, (*p*)-p65 and IL-1β were investigated by either RT-qPCR or immunobloting methods. In *in vitro* experiments, we treated (pre)-adipocytes with monocytes/macrophages and MAN, and investigated the changes of macrophages brought by pre-adipocytes co-culture. Generally, MAN restored the impaired fat anabolism in AIA rats, indicated by increased fat reservoir, leptin and adiponectin secretion, and PPAR-*γ* and SCD-1 expression. Meanwhile, it decreased circulating IL-1β and IL-6 levels, restored serological lipid profile changes, and relieved oxidative stresses, demonstrating potent therapeutic effects on AIA. AIA rats-derived monocytes inhibited mRNA PPAR-*γ* and SCD-1 expression in pre-adipocytes. Contrarily, MAN facilitated adipocyte differentiation *in vitro*, and increased free fatty acids production. It also significantly increased PPAR-*γ* and SCD-1 expression, which can be abrogated by PPAR-*γ* inhibitor T0070907. Similarly, lipopolysaccharide-primed macrophages inhibited PPAR-*γ* expression in the co-cultured pre-adipocytes, which was reversed by MAN. In the same co-culture system, lipopolysaccharide-induced inflammation was amplified by the co-existence of pre-adipocytes. More secretion of IL-1β and IL-6 and higher levels expression of COX-2, p-JNK, p-p65 and TLR4 were observed in lipopolysaccharide-treated macrophages when co-cultured by pre-adipocytes. The intensified inflammatory situation was eased by MAN. The treatment with pre-adipocytes culture medium achieved similar effects. Medium from lipopolysaccharide-treated adipocytes promoted IL-1β, IL-6 and MCP-1 production in separately cultured macrophages, and COX-2, p-JNK, p-p65 and TLR4 expression were increased at the meantime. MAN treatment on pre-adipocytes impaired these changes. It suggests that fat anabolism in AIA rats was deficient due to increased energy expenditure caused by inflammatory conditions. MAN restored fat metabolism homeostasis by up-regulating PPAR-*γ*, and reshaped secretion profile of adipocytes.

## Introduction

The pathogenesis of rheumatoid arthritis (RA) is yet to be fully understood, despite the advances in medical sciences. Until now, several factors have been identified as contributors to the occurrence of RA, including genetic background, environment pollution, infection, life habit, dietary, et cetera. Under certain circumstances, combination of these factors intensively activates auto reactive lymphocytes, which consequently initiate abnormal immune responses and perpetuate chronic inflammation (McInnes and Schett, 2011). Therefore, hyper-activation of lymphocytes, especially inflammatory CD4^+^ T cells serves as a basic pathological characteristic of RA. Meanwhile, monocytes/macrophages have been identified as another key player implicated in RA (Park et al., 2016). They are the dominant immune cells infiltrated into joints, and release majority of pro-inflammatory cytokines there. Additionally, they have the potential to activate osteoclasts and lymphocytes. Under such a context, manipulating monocytes/macrophages becomes a fascinating option for RA treatment. Many conventional anti-rheumatic drugs have been validated with notable inhibitory effects on these cells, and development of biological reagents targeting specific cytokines involved in their proliferation and maturation increasingly gains prominence (Alam et al., 2017). However, exact reasons leading to the excessive generation of inflammatory monocytes/macrophages in RA are still largely unknown.

It is obvious that functions of monocytes/macrophages are affected by metabolism status, and their polarization is tightly controlled by energy metabolism. Certain metabolic intermediates such as reactive oxygen species (ROS) and free fatty acids (FFAs) potently regulate their immune functions (Galván-Peña and O’Neill, 2014). Several metabolic regulators like sirtuin 1 (SIRT1) and peroxisome proliferator activated receptor gamma (PPAR-*γ*) are indispensable for immune homeostasis maintenance of these cells (Knethen and Brüne, 2003; Park et al., 2016). Hence, it is not surprising to find that many metabolic disorders are accompanied with persistent monocytes/macrophages-related inflammation (Lasselin and Capuron, 2014). Undoubtedly, disrupted energy metabolism contributes to the imbalanced polarization. In return, the accumulated inflammatory monocytes/macrophages aggravate metabolic disorders (Galván-Peña and O’Neill, 2014; Lasselin and Capuron, 2014). This pathological feedback can be observed in RA patients too. Obesity is a risk factor of inflammatory arthritis, and dyslipidemia is the most common complication of RA (García-Gómez et al., 2014; Arias de la Rosa et al., 2018). From this perspective, restoring metabolic homeostasis would benefit the therapy of RA. As such, compounds potent in regulating lipid metabolism could be potential anti-rheumatic candidates (Soulaidopoulos et al., 2018).

*α*-mangostin (MAN) is a naturally occurring xanthone derivative isolated from mangosteen pericarp. Previously, we found that MAN can effectively alleviate experimental arthritis in rats, and inhibit nicotinamide phosphoribosyltransferase (NAMPT) signaling activation in immune cells (Zuo et al., 2018a; Zuo et al., 2018b; Yang et al., 2019). Considering the crucial role of NAMPT in metabolism, we assumed that MAN treatment could extensively affect energy metabolism (Garten et al., 2015). This hypothesis is supported by many reports, and convincing evidences demonstrate that MAN possesses potent anti-obesity and anti-diabetic effects (Chen et al., 2018). Furthermore, it can suppress adipocytes differentiation in both *in vivo* and *in vitro* models (Nelli et al., 2013; Taher et al., 2015). All these clues implied that MAN could amplify its therapeutic effects on arthritis by disrupting the abnormal feedback between metabolism and immunity. In this study, we specifically investigated the impacts of MAN on fat metabolism in rats with adjuvant-induced arthritis (AIA), and attempted to decipher the relevance between its metabolism regulatory properties and anti-rheumatic potentials.

## Materials and Methods

### Chemicals and Reagents

Main reagents used in this study as well as their catalogue numbers were listed as below. Incomplete Freund’s adjuvant (IFA, R19016) and *Bacillus* Calmette-Guérin (BCG, R19021) were supplied by Rebio Scientific (Shanghai, China). The primary anti-SIRT1 (A17307), PPAR-*γ* (A19676), p-JNK (AP0473), JNK (A11119), p-p65 (AP0123), p65 (A11201), NAMPT (A0256), toll like receptor 4 (TLR4, A5258), cyclooxygenase-2 (COX-2, A1253), stearoyl-CoA desaturase 1 (SCD-1, A16429), CYP4A1 (A19662) and *β*-actin (AC026) antibodies were procured from AB clonal Technology (Wuhan, China). DMEM medium (C11995500BT), DMEM/F12 medium (C11330500BT), RPMI-1640 medium (C11875500BT), fetal bovine serum (FBS, 10099-141) and phosphate buffered saline (PBS, C10010500BT) were bought from Gibco (Grand Island, NY, United States). Cell counting kit-8 (CCK-8, C0037), bicinchoninic acid protein quantification kit (BCA, P0010), protein-loading buffer (P0015L), RIPA lysis buffer (P0013B), horseradish peroxidase/biotin-conjugated secondary antibody (A0208), together with nicotinamide (NAM, S1761) and resveratrol (ST1623) were provided by Beyotime Biotech (Nantong, Jiangsu, China). ELISA kits used for adiponectin (EK395-96), IL-1β (EK301B/3-96), IL-6 (EK306/3-96), and macrophage chemoattractant protein-1 (MCP-1, EK387-48) determination were purchased from Multi-Science (Hangzhou, Zhejiang, China). The biochemical quantification kits for total cholesterol (T-CHO, A111-1-1), low density lipoprotein cholesterol (LDL-C, A113-1-1), high density lipoprotein cholesterol (HDL-C, A112-1-1), malonaldehyde (MDA, A003-1-2), total superoxide dismutase (SOD, A001-1-1), reduced glutathione (GSH, A006-2-1) and nonesterified FFAs (A042-1-1) content assessment together with ELISA kit for leptin (H174) test were purchased from Jiancheng Bioengineering Institute (Nanjing, Jiangsu, China). Collagenase I (C8140), insulin (I8040), dexamethasone (D8040), 3-isobutyl-1-methylxanthine (IBMX, II0010), and Oil Red O staining kit (G1262) were bought from Solarbio (Beijing, China). Blood total triglyceride (TG, R02802) and glucose (R02402) quantification kits were purchased from Rayto (Shenzhen, Guangdong, China). TRNzol universal total RNA extraction reagent (DP424) and Luna universal qPCR master mix kit (M3003L) were purchased from Tiangen (Beijing, China) and New England BioLabs (Ipswich, MA, United States), respectively. Enhanced chemi-luminescence substrate reagent kit (ECL, WP20005) and ReverAid First Strand cDNA synthesis kit (K1622) were obtained from Thermo Fisher Scientific (Rockford, IL, United States). The selective PPAR-*γ* inhibitor T0070907 (T007, S2871) and MAN with the purity of 99% (6147-11-1) were procured from Selleck Chemicals (Shanghai, China) and BCYK Biotech (Nanjing, Jiangsu, China), respectively.

### Induction of Adjuvant-Induced Arthritis in Rats and Treatments

Male Sprague Dawley rats (180 ± 10 g) were supplied by Qinglongshan Laboratory Animal Company (Nanjing, Jiangsu, China). The animals were housed in a specific pathogen free (SPF) laboratory with standard environmental conditions. All the rats were fed with sterile rodent chow and water ad libitum. After 7 days acclimatization, AIA was induced in all the rats except for normal healthy controls. Prior to this procedure, heating-inactivated BCG was carefully grinded in IFA, and subsequently homogenized with the same volume of water to obtain complete Freund’s adjuvant (CFA, 20 mg/ml). On day 0, an intradermal injection of 0.1 ml CFA was carried out on the right hind paw, which was followed by a boost CFA injection at the base of tail 7 days later. Thereafter, the rats were divided into three groups with six rats each, and received different oral treatments:

Group 1: Normal healthy control (normal) administered with 0.5% sodium carboxymethyl cellulose solution (CMC-Na); Group 2: AIA model control (AIA) administered with 0.5% CMC-Na; Group 3: MAN-treated AIA rats (MAN + AIA) administered with MAN (dispersed in 0.5% CMC-Na by the aid of ethanol and tween-80).

The therapeutic dose of MAN was designated as 50 mg/kg, as it can result in effective concentrations in both blood and tissues (Xu et al., 2017). Also, MAN at this dose can effectively cure experimental arthritis (Yang et al., 2019). The treatment duration was 30 days, and body weight of all the rats was periodically recorded. All the animal experiment procedures were performed in accordance with the national institutes of health guide for the care and use of laboratory animals (NIH Publications No. 8023, revised 1978) and the approval for animal studies in this study was obtained from the Ethics Committee of Wannan Medical College.

### Clinical Manifestation Evaluation

**FIGURE 1:**
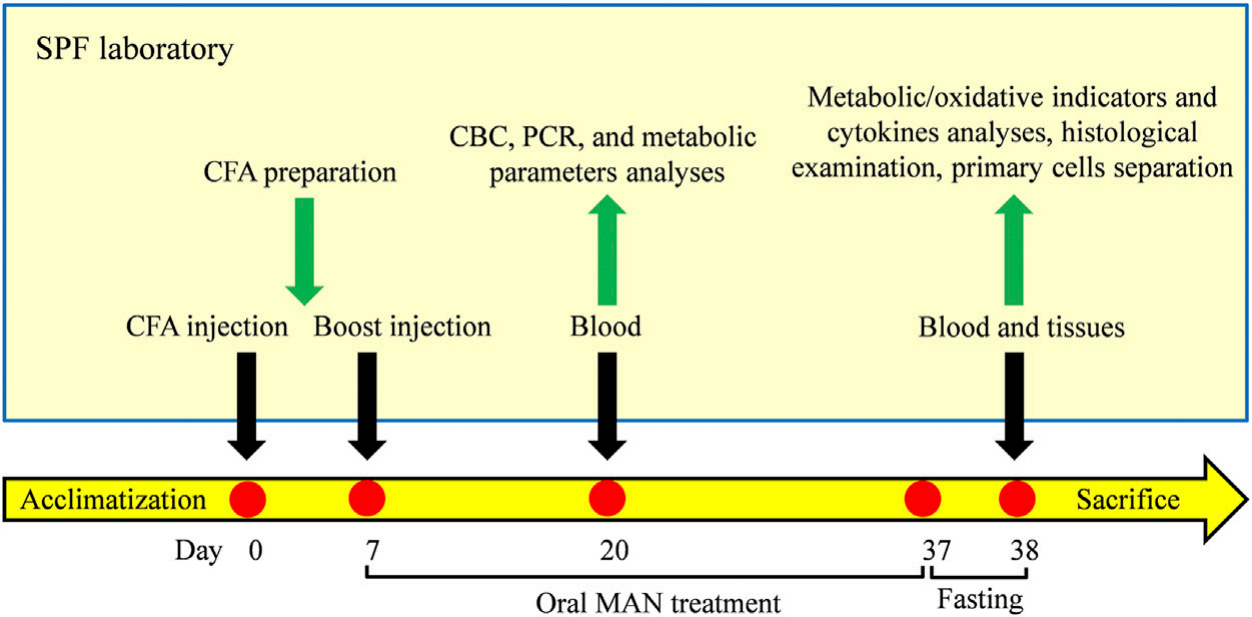
Experimental flow of the *in vivo* experiment.

On day 20, the anticoagulation blood of rats was collected from fossa orbitalis vein. Complete blood cell counts (CBC) was performed with the aid of a hematology analyzer (Prokan, Shenzhen, China). Blood glucose and TG levels were determined using corresponding kits in accordance with the manufactures’ instructions on an automatic biochemical analyser. After the lysis of red cells, total blood white cells were obtained after centrifugation. The expression of mRNA NAMPT, SIRT1, PPAR-*γ* and IL-1β in these cells were investigated by quantitative reverse transcription polymerase chain reaction (RT-qPCR) method. By the end of observational period, all the rats were fasted overnight. The next day, blood samples were collected from the abdominal aorta upon the anesthetization with chloral hydrate. The serum obtained was used for the quantification of T-CHO, LDL-C, HDL-C, FFAs, MDA, SOD, GSH, leptin, adiponectin, IL-1β, and IL-6 using appropriate kits strictly in accordance with the manufacturers’ protocols. One rat from each group was dissected under sterile conditions to separate primary cells. Unilateral fat pad from left kidney of the rest rats were removed and weighed after sacrifice. Portion of the tissues was subjected to hematoxylin-eosin (H&E) staining-based histological examination. The remaining fat samples were used for the extraction of protein and RNA. Expression of mRNA NAMPT, SIRT1, PPAR-*γ* and IL-1β was assessed by RT-qPCR method, and expression of protein NAMPT, SIRT1, PPAR-*γ*, SCD-1 and CYP4A1 was investigated by immunoblotting approach. The *in vivo* experimental flow is illustrated in Figure 1.

### Cell Preparation and Culture

To obtain pre-adipocytes, perirenal fat tissues were cut into small pieces (1 mm^3^) in a sterile environment, which were then digested with 0.1% collagenase I at 37°C for 60 min. Thereafter, the collagenase was neutralized with FBS. The resulting mixture was filtered with a 200 mesh sieve. Finally, the unicellular suspension was obtained, and cells within were collected after centrifugation at 1,500 rpm for 10 min, which were re-suspended in DMEM/F12 medium containing 10% FBS. The culture medium was replaced every other day, and the cells at exponential growth stage from the 3rd to 6th passages were taken as pre-adipocytes (Zuo et al., 2021). Immediately after the sacrifice, PBS was intraperitoneally injected into the abdominal cavity. The abdomen was then gently rubbed for 10 min, and the liquid was withdrawn using a glass syringe. Macrophages obtained after centrifugation were washed with PBS and subsequently cultured in complete RPMI-1640 medium. Monocytes within anticoagulation blood were separated with gradient centrifugation method using a commercial kit (Solarbio, P6700), and cultured in DMEM medium. Macrophages and monocytes were used without further passage. All these cells were cultured at 37°C in humidified air with 5% CO_2_.

### Assessment of Effects of *α*-mangostin on (pre)-Adipocytes

The potential cytotoxicity of MAN on pre-adipocytes was assessed using CCK-8 reagent. Pre-adipocytes were seeded in 96-well plates at a density of 5,000 cells/well. After overnight culture, the cells were treated with MAN at various concentrations for 24 h. Subsequently, the supernatant was replaced by 100 μl of fresh medium, and 10 μl of CCK-8 solution was added. After a further incubation for 4 h, the optical density for each well was measured at 450 nm, which was then used to calculate the viability of treated cells by taking untreated cells as a reference.

To induce adipocyte differentiation, pre-adipocytes were cultured with complete medium supplemented with 0.5 mM IBMX, 1 μM dexamethasone, and 10 μg/L insulin for 3 days. Then, the supernatant was replaced by DMEM/F12 medium containing insulin and FBS, and cells were further cultured for two additional days (Cheng et al., 2016). Thereafter, the cells were maintained in normal medium in the presence of MAN or not for 2 days. Adipocytes differentiation status was evaluated by FFAs production and intracellular lipid droplet distribution. To stain intracellular lipid droplets, cells were fixed in 10% neutral formalin, and then soaked in 60% isopropanol for 5 min, which was followed by Oil Red O staining for 20 min. Finally, hematoxylin staining was carried out. Some cells receiving the same treatments were harvested for PCR and immunoblotting analyses to assess the expression of NAMPT, SIRT1, PPAR-*γ* and SCD-1. To confirm the signaling accounting for MAN-induced metabolic changes, some pre-adipocytes were treated with MAN at an optimized concentration in combination with T007 (10 μM), NAM (5 mM) or resveratrol (25 μM), and expression of mRNA SIRT1, PPAR-*γ* and SCD-1 in these cells were subsequently analyzed.

### Cell Co-treatment

We investigated the impact of AIA immune environment on adipocytes differentiation using a co-culture system. (Pre)-adipocytes and different rats-derived monocytes (normal healthy controls, AIA models, MAN-treated AIA rats) were seeded in the lower and upper chambers of transwell (with pores of 0.4 μm), respectively. Twenty-four hours later, (pre)-adipocytes were collected for the analysis of mRNA PPAR-*γ* and SCD-1 expression.

To further clarify the interaction between pre-adipocytes and macrophages under different conditions, the two types of cells were co-cultured in the presence of LPS and MAN. The pre-adipocytes seeded in down chambers of trans well were allowed to attach and then treated with MAN (4 μg/ml), while the attached peritoneal macrophages in the upper chamber were stimulated with LPS (400 ng/ml). After a 24 h co-culture, all the cells were harvested. CYP4A1, SIRT1, PPAR-*γ*, and SCD-1 expression in pre-adipocytes and IL-1β, iNOS, p-JNK, p-p65, and COX-2 expression in macrophages were analyzed by either PCR or immunoblotting method. Levels of IL-1β and IL-6 from the upper chamber were determined by ELISA kits.

Subsequently, we stimulated macrophages with medium collected from pre-adipocytes culture system. The pre-adipocytes seeded in 6-wells plates were treated by MAN in the presence of LPS or not for 24 h. The medium was then obtained, which was mixed with equal amount of fresh complete medium and used for normal macrophages culture. Twenty four hours later, the macrophages were harvested for PCR and immunoblotting analyses, and their culture medium was collected for IL-6, IL-1β and MCP-1 content assessment.

### RT-qPCR and Immunoblotting Experiments

To analyze mRNA expression, total RNA in both tissues and cells samples were extracted using Trizol reagent, which was subsequently reverse-transcribed into cDNA using the synthesis kit. Samples with equal amount of cDNA were subjected to RT-qPCR procedure on an ABI 7500 quantitative PCR instrument. Relative expression of specific gene was assessed based on 2^−ΔΔ^Ct calculation using *β*-actin as the internal reference (Wang et al., 2021). The sequences of primers used in this study are included in Supplementary Data1.

In immunoblotting assays, tissues and cells samples were lyzed in pre-chilled RIPA buffer (containing 1% proteases and phosphatase inhibitors). The supernatant obtained from a high-speed centrifugation was mixed with loading buffer, and denatured in boiling water for 5 min. Thereafter, the quantified proteins were separated by sodium dodecyl sulfate-polyacrylamide gel electrophoresis and transferred onto blotting membranes. The protein-loading membranes were subsequently blocked with 5% skim milk, which was followed by primary antibodies incubation at 4°C overnight. After appropriate secondary antibodies treatment and interval washing, signals were developed using an ECL kit and examined on a Tanon 5,200 system (Bio-Tanon, Shanghai, China).

### Statistical Analysis

The data were presented as mean ± standard deviation (SD). Most of statistical analyses related to animal samples were based on sextuplicate, while five samples were included in fat tissue-related analyses, since one rat from each group were used to prepare primary cells separately. Experiments *in vitro* were performed in triplicate or quadruplicate. Semi-quantification of immunoblotting assays was achieved by the aid of ImageJ (version 1.52a, NIH, Bethesda, MD, United States). Statistical differences among groups were analyzed by GraphPad Prism 8.0 (GraphPad Software, Cary, NC, United States) using one-way analysis of variance coupled with Tukey post hoc test. *p* values less than 0.05 were considered statistically significant.

## Results

### α-mangostin Possibly Altered Energy Metabolism of Adjuvant-Induced Arthritis Rats

**FIGURE 2:**
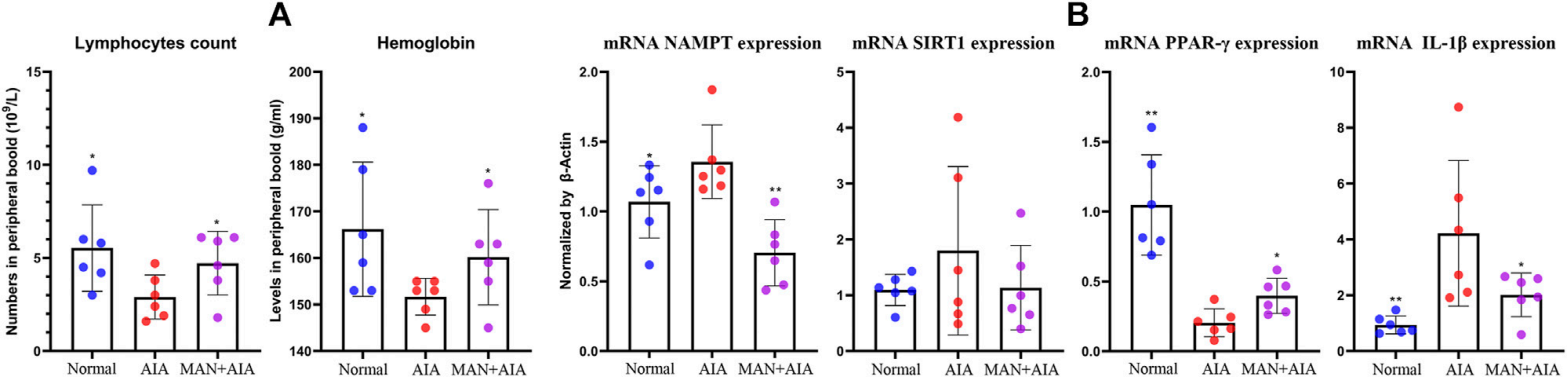
Effects of MAN on AIA rats during the secondary inflammation phase. **(A)** changes of lymphocytes and hemoglobin counts; **(B)** expression of mRNA NAMPT, SIRT1, PPAR-*γ* and IL-1β in blood white cells. Statistical significance: *p <0.05 and *p < 0.01 compared with AIA rats (*n* = 6).

The anti-rheumatic efficacy of MAN has been systematically investigated in previous researches (Zuo et al., 2018a; Zuo et al., 2018b). In this study, we mainly investigated its metabolism regulatory effects on AIA rats, and thereby no positive drug was included. AIA usually develops 14 days after the 1st CFA injection. The inflammatory manifestations reach the peak at approximately 20 days after AIA induction, and are ameliorated spontaneously thereafter (Zuo et al., 2018b). Under this context, some pharmacological properties of MAN could be hardly observed by the end of experiment. Hence, we collected peripheral blood on day 20 and analyzed some basic clinical parameters. In CBC analysis, AIA rats showed decreased counts of lymphocytes and hemoglobin, which was similar to the changes in RA patients (Bowman, 2002). Treatment with MAN increased both lymphocytes and hemoglobin counts, suggesting the alleviated AIA severity (Figure 2A). RT-qPCR analysis performed on blood white cells demonstrated that the expression of mRNA NAMPT and IL-1β in AIA rats were significantly elevated, indicating an inflammatory micro-environment and up-regulated polarization of inflammatory monocytes (Liu et al., 2012; Garten et al., 2015). As a downstream target of NAMPT, SIRT1 expression was slightly increased. Of note, a dramatic decrease of PPAR-*γ* expression was observed in AIA rats. All these changes were restored in MAN-treated AIA rats (Figure 2B). Regulation on NAMPT and PPAR-*γ* did not only indicate the anti-inflammatory properties of MAN, but also promised profound metabolic changes. Indeed, obvious metabolic alteration occurred. Levels of glucose and TG in peripheral blood were reduced in AIA rats during the peak of secondary inflammation, which were reversed by MAN treatment (Table 1).

**TABLE 1:** Metabolic parameters detected in peripheral blood in rats.

	Normal healthy rats	AIA model rats	MAN-treated AIA rats
TG (mM)	1.00 ± 0.57	0.69 ± 0.43	0.81 ± 0.52
Glucose (mM)	4.69 ± 1.25^*^	2.39 ± 1.31	6.31 ± 2.32^*^
T-CHO (mM)	1.98 ± 0.26^**^	2.51 ± 0.53	1.82 ± 0.31^**^
HDL-C (mM)	1.49 ± 0.14	1.11 ± 0.27	1.35 ± 0.28
LDL-C (mM)	1.74 ± 0.09	2.26 ± 0.43	1.77 ± 0.24
Nonesterified FFAs (μM)	210.7 ± 118.8	232.4 ± 33.9	100.2 ± 18.0^**^

Levels of TG and glucose were determined in blood collected on day 20, and the rest analyses were performed on samples obtained after the sacrifice of rats on day 38. The data were presented as mean ± SD, *n* = 6. The statistical difference between AIA models and normal healthy/MAN-treated AIA rats was considered significant when *p < 0.05 and **p < 0.01.

### α-mangostin Attenuated Pathological Changes in Fat Tissues in Adjuvant-Induced Arthritis Rats

**FIGURE 3:**
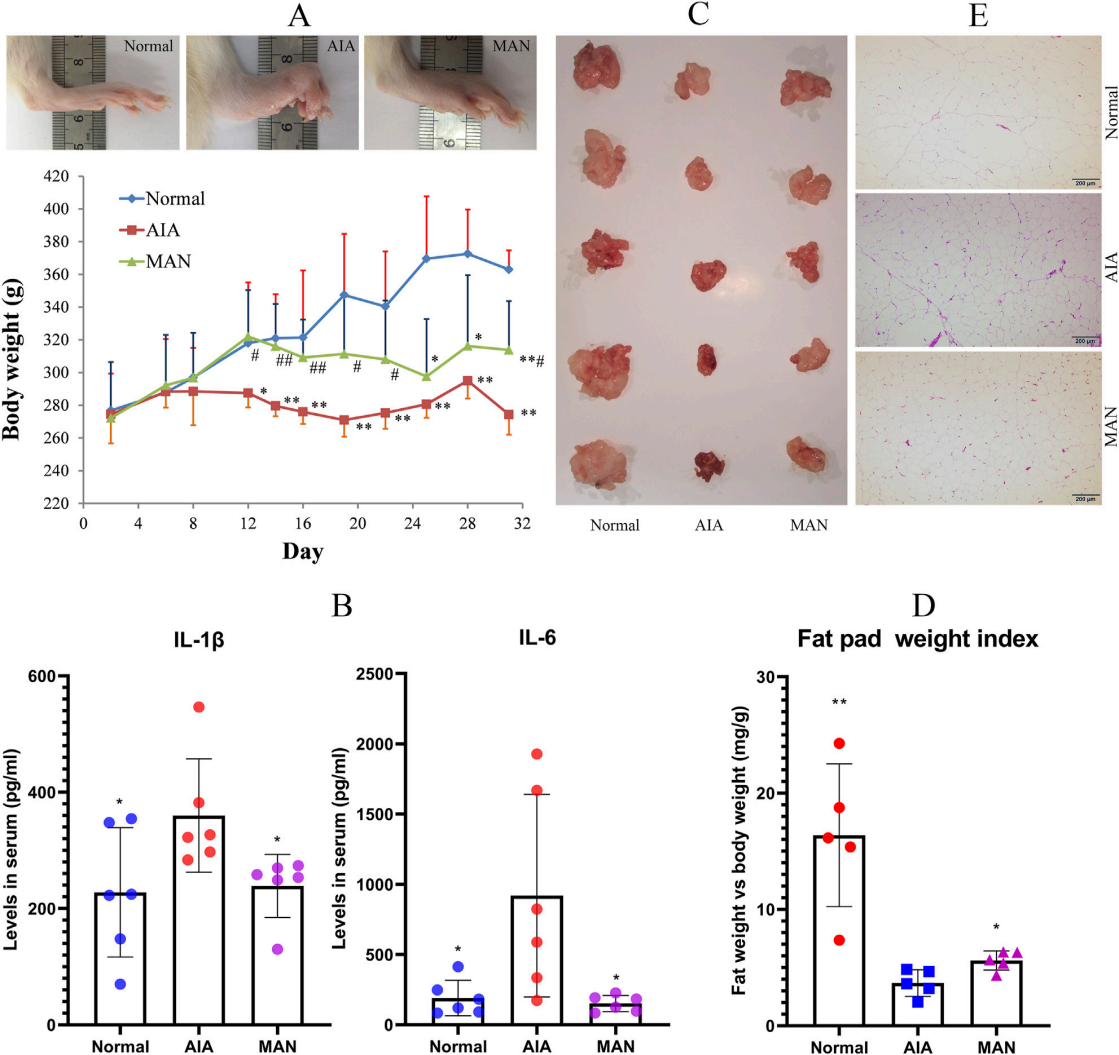
MAN restored fat reservoir loss in AIA rats. **(A)** local inflammation in paws and body weight changes of rats; **(B)** levels of IL-1β and IL-6 in rat serum; **(C)**, images of perirenal fat pad; **(D)** relative weight index of fat pad; **(E)** histological examination of WAT. Statistical significance: *p < 0.05 and **p < 0.01 compared with AIA rats [*n* = 6 in image **(A,B)**, *n* = 5 in image **(D)**].

To maximize AIA-related body weight changes, the rats were immunized by a second CFA injection. Severe paw edema occurred in all CFA-immunized rats since day 14, which was accompanied with obvious body weight loss. The body weights of AIA rats were constantly lower than healthy controls thereafter, but this situation was spontaneously ameliorated during the later stage. Whereas, obvious restoration of body weight loss was observed in MAN-treated AIA rats (Figure 3A). Subsequently, we determined levels of IL-1β and IL-6 in serum, two important mediators involved in white adipose tissue (WAT)-related inflammation (Lasselin and Capuron, 2014). The results showed that MAN efficiently suppressed the abnormal increase of IL-1β and IL-6 in AIA rats (Figure 3B). As illustrated in Figure 3C, fat reservoir in AIA rats was greatly depleted, and MAN treatment attenuated this trend. The relative fat weight index adjusted by body weight further confirmed this finding (Figure 3D). H&E staining-based histological examination revealed shrunken adipocytes size in AIA rats. Meanwhile, vivid inflammatory infiltration and angiogenesis occurred. Although MAN did not effectively restore the adipocytes size, the inflammatory manifestation was largely eased (Figure 3E).

### α-mangostin Reshaped Lipid Metabolism in Adjuvant-Induced Arthritis Rats

**FIGURE 4:**
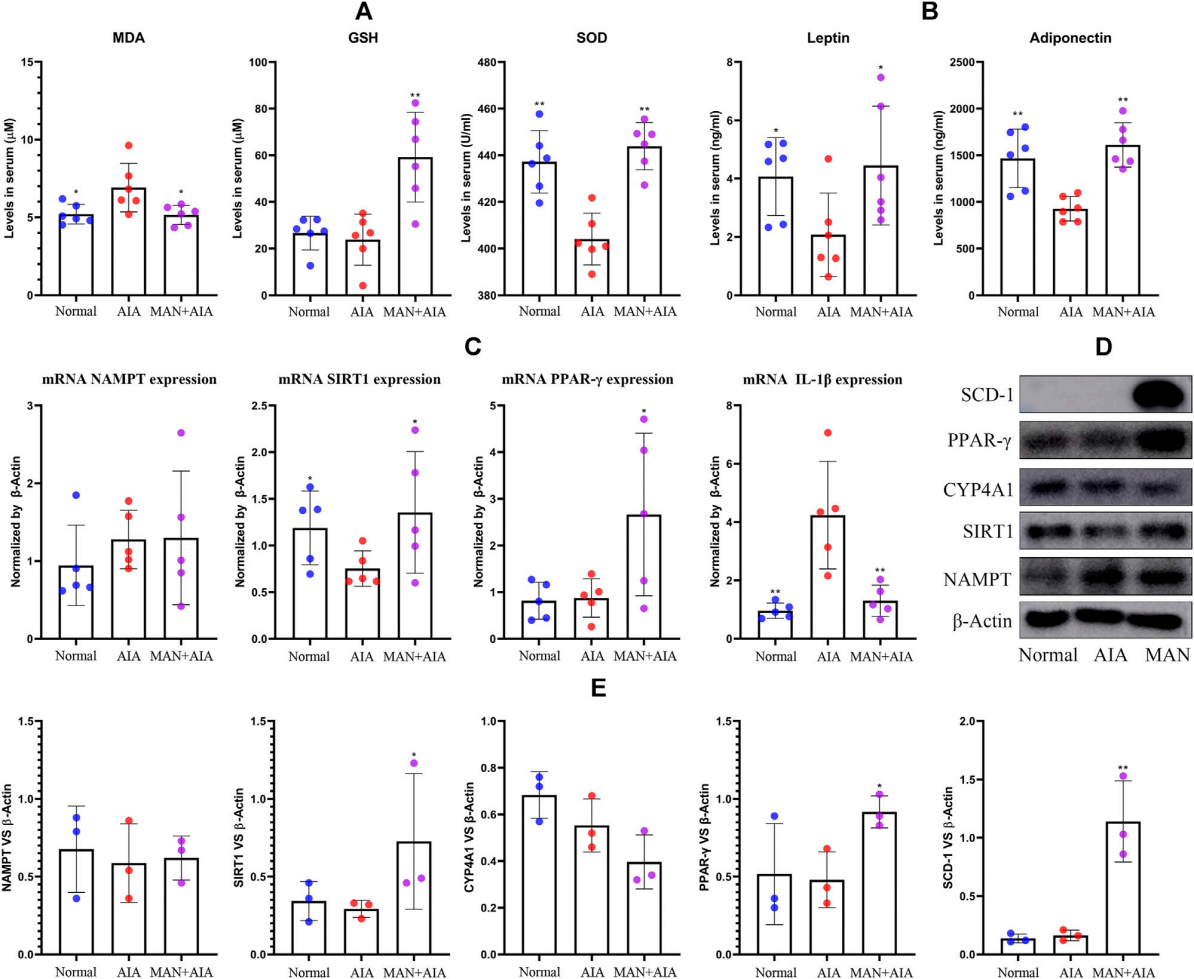
Changes of lipid metabolism-related parameters in rats. **(A)** levels of oxidative stress indicators (MDA, GSH, SOD) in serum; **(B)** levels of leptin and adiponectin in serum; **(C)** expression of mRNA NAMPT, SIRT1, PPAR-*γ* and IL-1β in WAT; **(D)** expression of protein NAMPT, SIRT1, CYP4A1, PPAR-*γ* and SCD-1 in WAT; **(E)** quantification result of assay **(D)**. Statistical significance: *p < 0.05 and **p < 0.01 compared with AIA rats [*n* = 6 in image **(A,B)**, *n* = 5 in image **(C)**, *n* = 3 in image **(E)**].

Due to the spontaneous amelioration of AIA, the concomitant metabolic changes became not very significant when the rats were sacrificed, but the dyslipidemia can be still observed. Levels of T-CHO, LDL-C and nonesterified FFAs were increased, while HDL-C was decreased in AIA rats. However, only the change of T-CHO was statistically significant. Generally, the disordered lipid profile was restored by MAN treatment (Table 1). Of note, the level of nonesterified FFAs in AIA rats was reduced from 232.4 to 100.2 μM upon MAN treatment. Because lipid metabolism is closely related to endogenous oxidative stresses, we analyzed some oxidative stress biomarkers. Although GSH remained unchanged, significant increase in MDA content and decrease in SOD activity were observed in AIA rats. MAN significantly elevated GSH levels, and restored the abnormal changes of MDA and SOD (Figure 4A). Changes of adipokines further revealed the altered fat metabolism *in vivo*. Levels of leptin and adiponectin in serum were reduced in AIA models, while MAN reversed these declines (Figure 4B). We thought NAMPT was the key to metabolism regulatory properties of MAN (Tao et al., 2018; Yang et al., 2019). However, RT-qPCR analysis performed on WAT found that there was no significant change in NAMPT expression in either AIA models or MAN-treated rats. As we found significant changes of NAMPT on day 20, these clues preliminarily suggest that inflammation exerts diversified effects on metabolism at different stages. Interestingly, the expression of its downstream SIRT1 was largely impaired in AIA rats, while MAN up-regulated it. Similar to results from analysis performed on blood white cells mentioned above, MAN significantly increased PPAR-*γ* expression in WAT, by almost 3-fold magnitude. Probably due to the anti-inflammatory nature of SIRT1 and PPAR-*γ*, their increase was synchronized with dramatic decrease of IL-1β expression (Figure 4C). Immunoblotting assays confirmed the regulatory effects of MAN on SIRT1 and PPAR-*γ* (Figure 4D). Notably, the expression of SCD-1, a downstream of PPAR-*γ* and an important enzyme for fat anabolism was significantly increased by MAN. Quantification of these results obtained similar conclusion to that from PCR analysis (Figure 4E). Barely changed CYP4A1 expression suggested that MAN had little effect on fat oxidation. All the uncropped images of immunoblotting assays in this study were included in Supplementary Data2. It can be concluded that adipocytes differentiation within WAT was impaired in AIA rats, which was indicated by decreased fat reservoir and adipokines secretion. The declined PPAR-γ expression on day 20 also supported this. This defect eventually resulted in accumulated circulating lipids (T-CHO, LDL-C and FFAs). On the other side, fat oxidation in AIA rats was accelerated, reflecting in deficiency of anti-oxidative capacity and accumulated MDA. Generally, MAN exhibited potent effects against AIA-caused metabolic abnormalities. Considering the crucial role of PPAR-*γ* in WAT development, we mainly emphasized this signaling thereafter.

### α-mangostin-Induced Proliferator Activated Receptor-*γ* Up-Regulation Facilitated Adipocytes Differentiation

**FIGURE 5:**
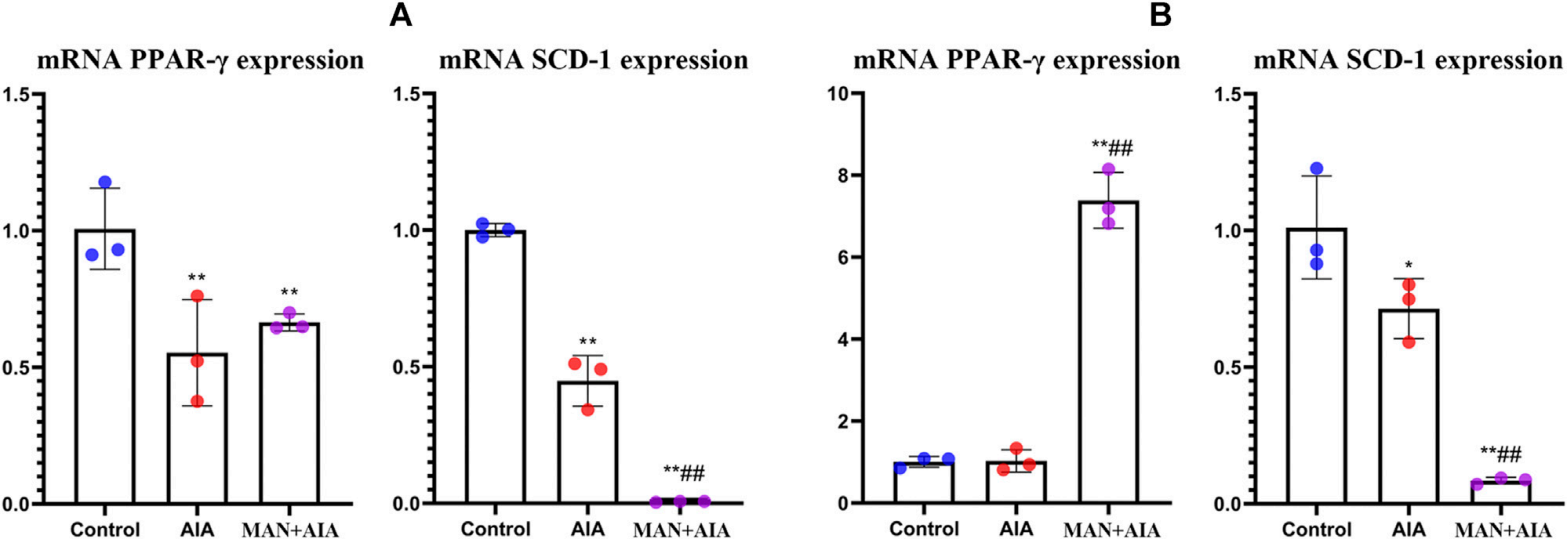
Effects of different immune conditions on (pre)-adipocytes. **(A)** expression of mRNA PPAR-*γ* and SCD-1 in pre-adipocytes co-cultured with monocytes obtained from normal healthy, AIA control or MAN-treated AIA rats; **(B)** the replicate of assay **(A)** by using adipocytes instead of pre-adipocytes. Statistical significance: *p < 0.05 and **p < 0.01 compared with (pre)-adipocytes co-cultured with normal monocytes; ^#^ *p* < 0.05 and ^##^ *p* < 0.01 compared with (pre)-adipocytes co-cultured with AIA rats-derived monocytes (*n* = 3).

AIA rats-derived monocytes suppressed PPAR-*γ* and SCD-1 expression in pre-adipocytes (Figure 5A). In the case of adipocytes, the situation was a bit different. No change of PPAR-*γ* expression was induced by AIA monocytes. A plausible explanation for this is that PPAR-*γ* is the key regulator for pre-adipocyte but not adipocyte (Taher et al., 2015). Consequently, it suggested that pre-adipocytes should be the priority in following experiments. Comparatively, (pre)-adipocytes co-cultured with monocytes from MAN-treated AIA rats showed higher level of mRNA PPAR-*γ*, but SCD-1 expression was further decreased (Figure 5B). This result is confusing, because SCD-1 expression fluctuated in accordance to PPAR-*γ* in AIA rats. It implied that SCD-1 expression was not exclusively determined by PPAR-*γ*. Indeed, nutrition status, hormonal levels, non-coding RNA, and transcription factors are all involved in its regulation (Liu, 2009). Thereby, elucidating exact impacts of MAN on the micro-environment in AIA rats could be the key to address this paradox.

**FIGURE 6:**
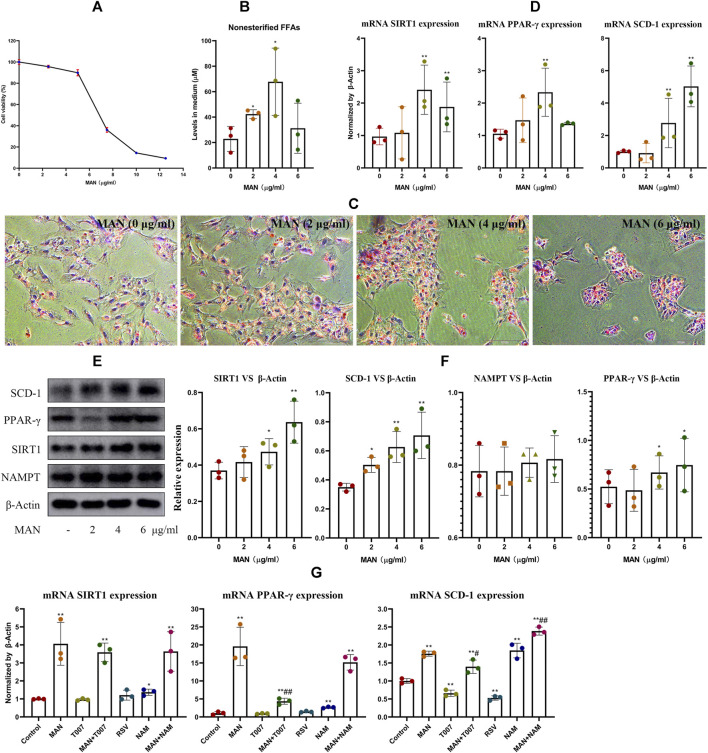
MAN promoted adipocyte differentiation *in vitro*. **(A)** cytotoxicity of MAN on pre-adipocytes at various concentrations; **(B)** levels of non-esterified FFAs in medium supernatant from pre-adipocytes receiving MAN treatments or not; **(C)** morphology observation of cells from assay **(B)** by the aid of Oil Red O staining; **(D)** expression of mRNA SIRT1, PPAR-*γ* and SCD-1 in cells from replicate experiment **(B)**; **(E)** expression of protein NAMPT, SIRT1, PPAR-*γ* and SCD-1 in cells from replicate experiment **(B)**; **(F)** quantification result of assay **(E)**; **(G)** expression of mRNA SIRT1, PPAR-*γ* and SCD-1 in pre-adipocytes treated by various combination of chemicals (MAN, resveratrol, T007 and NAM). Statistical significance: *p < 0.05 and **p < 0.01 compared with untreated cells; ^#^ *p* < 0.05 and ^##^ *p* < 0.01 compared with MAN-treated pre-adipocytes (*n* = 3).

CCK-8 assay revealed that MAN treatment for 24 h had significant cytotoxic effect on pre-adipocytes at concentrations above 6 μg/ml (Figure 6A). As a result, 2, 4 and 6 μg/ml were adopted as the low, medium and high treatment concentrations. Generally, MAN boosted FFAs synthesis in adipocytes (Figure 6B). Meanwhile, it promoted adipocyte differentiation as indicated by darker Oil Red O staining, especially at 4 and 6 μg/ml, irrespective of cell population variations (Figure 6C). Consistently, MAN treatments increased the expression of mRNA PPAR-*γ* and SCD-1 in adipocytes. Also, SIRT1 expression was increased (Figure 6D). The most significant changes induced by MAN concerning the levels of FFAs, PPAR-*γ*, SIRT1 were achieved at 4 μg/ml, probably due to the impaired cell viability at 6 μg/ml. Therefore, 4 μg/ml was adopted as the optimized concentration afterward. In immunoblotting assay, NAMPT still remained unchanged under MAN stimulus, while the expression of PPAR-*γ* and SCD-1 as well as SIRT1 was significantly increased (Figures 6E,F). Since both SIRT1 and PPAR-*γ* are involved in fat metabolism, we further investigated their roles in above metabolic changes. PPAR-*γ* inhibitor T007 did not affect SIRT1 and PPAR-*γ* levels, but significantly reduced SCD-1 expression. SIRT1 inhibitor NAM promoted the expression of PPAR-*γ* and SCD-1, while SCD-1 expression was efficiently suppressed by SIRT1 agonist resveratrol. The combined stimulus of T007 impaired the effects of MAN on SCD-1 expression in pre-adipocytes, and the addition of NAM achieved the opposite outcome (Figure 6G). These facts validated the reciprocal negative regulation between SIRT1 and PPAR-*γ*, and confirmed that MAN promoted adipocytes differentiation by up-regulating PPAR-*γ* signaling.

**FIGURE 7:**
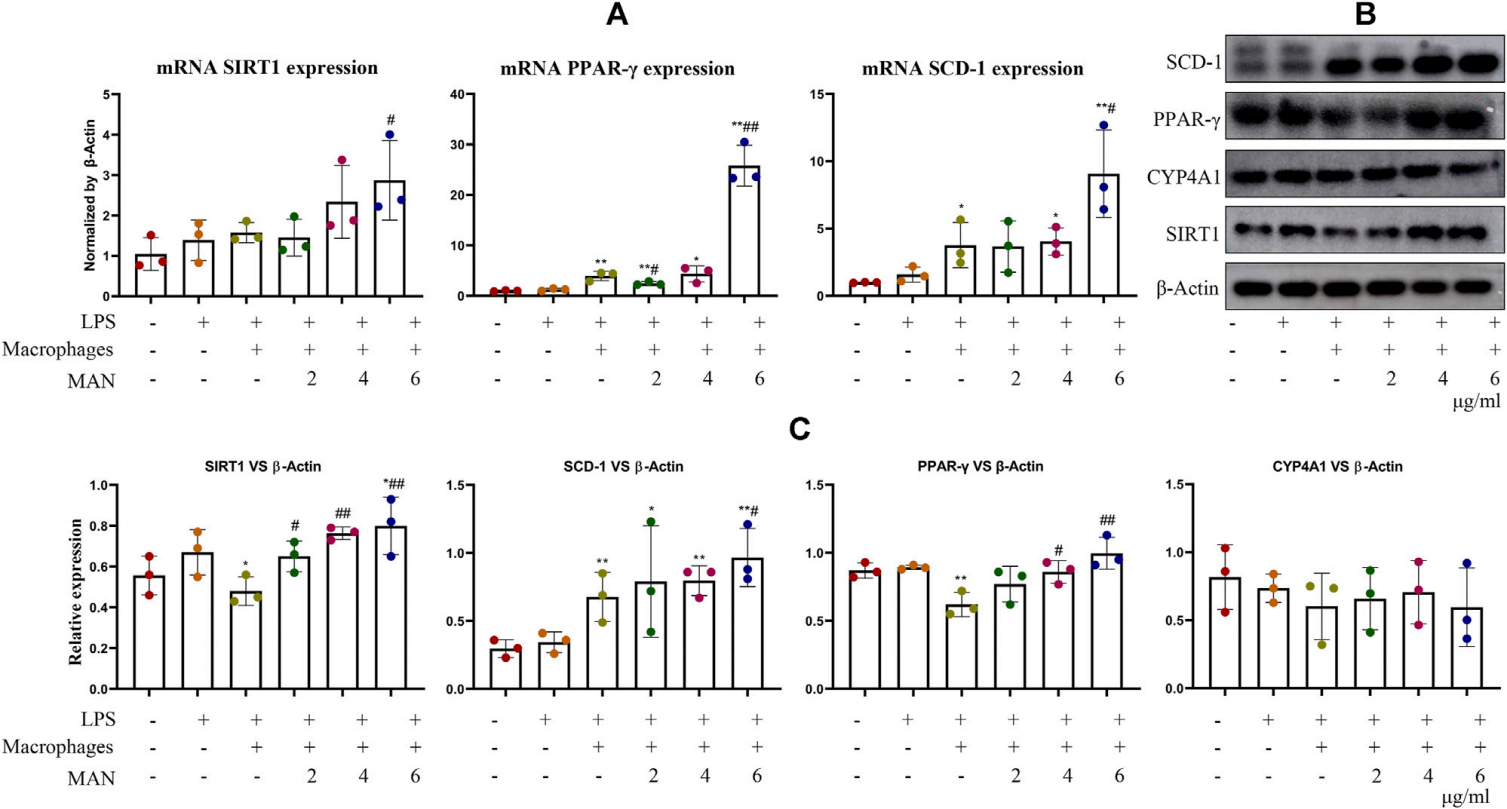
Effects of MAN on pre-adipocytes co-cultured with inflammatory macrophages. **(A)** expression of mRNA SIRT1, PPAR-*γ* and SCD-1 in pre-adipocytes receiving MAN treatments or not in the co-existence of LPS-primed macrophages; **(B)** expression of protein SIRT1, PPAR-*γ* and SCD-1 in pre-adipocytes receiving the same treatments with assay **(A)**; **(C)** quantification result of experiment **(B)**. Statistical significance: *p < 0.05 and **p < 0.01 compared with LPS-stimulated pre-adipocytes without the presence of macrophages; ^#^ *p* < 0.05 and ^##^ *p* < 0.01 compared with pre-adipocytes co-cultured with LPS-primed macrophages (*n* = 3).

To clarify the combined impact of direct MAN stimulus and immune factors on WAT, we investigated changes of MAN-treated pre-adipocytes in the presence of inflammatory macrophages. LPS-primed macrophages promoted mRNA SIRT1, PPAR-*γ* and SCD-1 expression in pre-adipocytes. MAN even further increased their levels (Figure 7A). In contrast to findings obtained from PCR analysis, results from the immunoblotting assay indicated that inflammatory macrophages down-regulated the expression of protein PPAR-*γ* and SIRT1 in pre-adipocytes (Figure 7B). These results demonstrated the dynamic immune re-balancing under inflammatory conditions (Hackett et al., 2008). Acute inflammatory stimulus promoted the expression of anti-inflammatory mediators including PPAR-*γ* and SIRT1 to quench the flame. However, this attempt may fail under intense inflammation, because their mRNA can be destabilized (Jennewein et al., 2010; Zhang et al., 2019). Once again, we noticed that the levels of SIRT1, PPAR-*γ* and SCD-1 were increased by MAN. Meanwhile, CYP4A1 expression remained unchanged (Figure 7C). Overall evidence suggested that MAN can facilitate PPAR-*γ*-controlled adipocyte differentiation through direct stimulus and immune changes.

### α-mangostin Disrupted Inflammatory Immune-Metabolism Feedback by Targeting Pre-adipocytes

**FIGURE 8:**
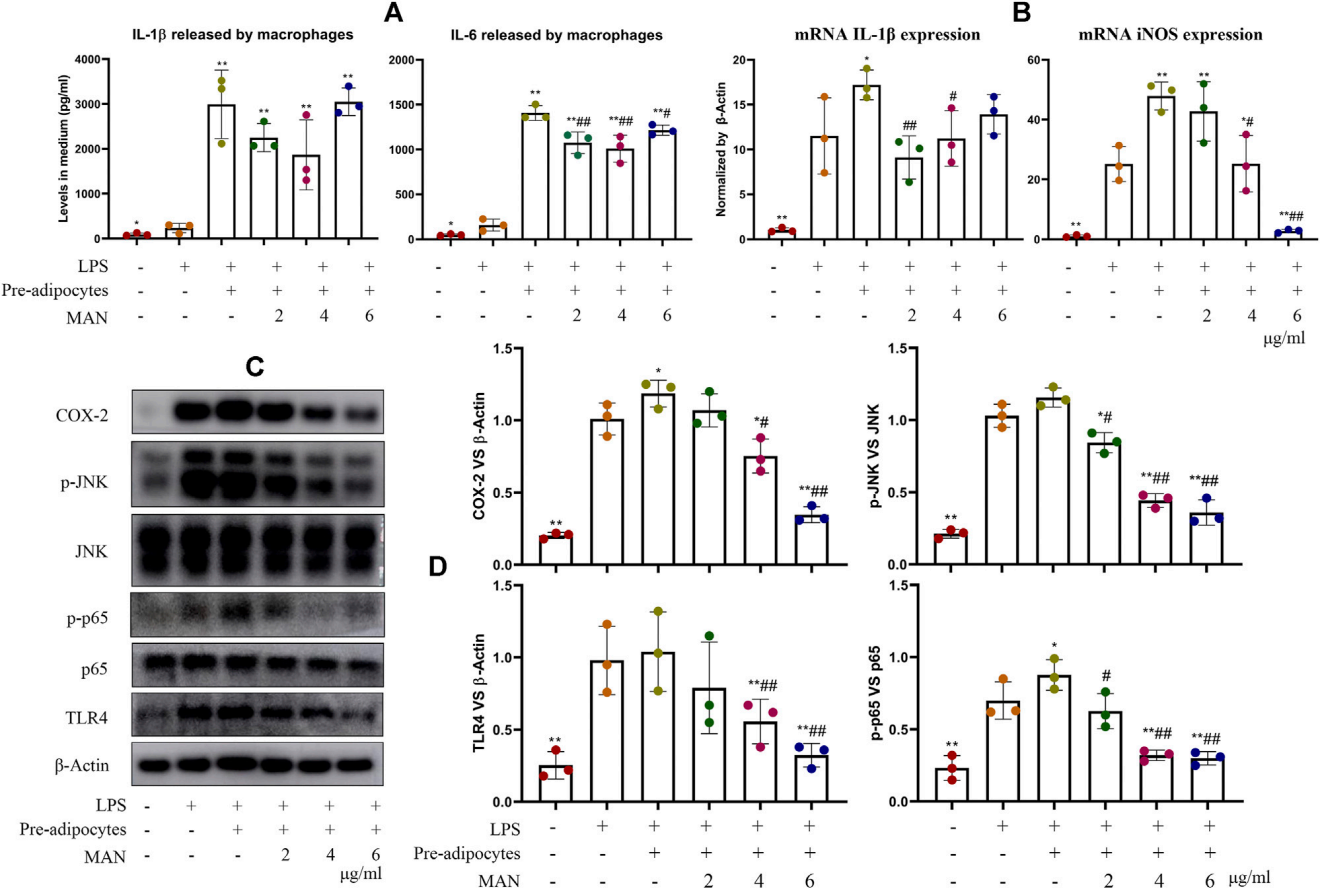
Effects of MAN on inflammatory macrophages co-cultured with pre-adipocytes. **(A)** levels of IL-1β and IL-6 in medium from macrophages culture chamber; **(B)** expression of mRNA IL-1β and iNOS in LPS-primed macrophages co-cultured with pre-adipocytes in the presence of MAN or not; **(C)** expression of protein TLR4, p65, p-p65, JNK, p-INK and COX-2 in above macrophages; **(D)** quantification result of assay **(C)**. Statistical significance: *p < 0.05 and **p < 0.01 compared with LPS-stimulated macrophages; ^#^ *p* < 0.05 and ^##^ *p* < 0.01 compared with LPS-primed macrophages in the presence of untreated pre-adipocytes (*n* = 3).

Afterward, we analyzed macrophages co-cultured with pre-adipocytes to decipher the immune implication of above metabolic changes. IL-1β and IL-6 contents in the medium from LPS-stimulated macrophages were significantly higher than vehicle control (234.4 vs. 92.6 pg/ml for IL-1β, 158.0 vs. 60.0 pg/ml for IL-6). LPS-induced inflammation was further greatly amplified by the co-culture of pre-adipocytes. Levels of IL-1β and IL-6 were increased to 2992.5 and 1359.5 pg/ml, respectively (Figure 8A). MAN attenuated this pro-inflammatory cytokines increase. The up-regulation of IL-1β and iNOS as well as their upstream TLR4, COX-2, p-JNK and p-p65 in LPS-treated macrophages were similarly reinforced by pre-adipocytes (Figures 8B,C). MAN potently suppressed expression of mRNA IL-1β and iNOS in these macrophages. Notably, iNOS levels almost reached the basal level in the presence of MAN at 6 μg/ml (Figure 8B). Expression of protein TLR4, COX-2, p-JNK and p-p65 in the macrophages were down-regulated by MAN similarly (Figure 8D). As macrophages were not directly stimulated by MAN, we assumed that its anti-inflammatory effects were mainly mediated by pre-adipocytes changes.

**FIGURE 9:**
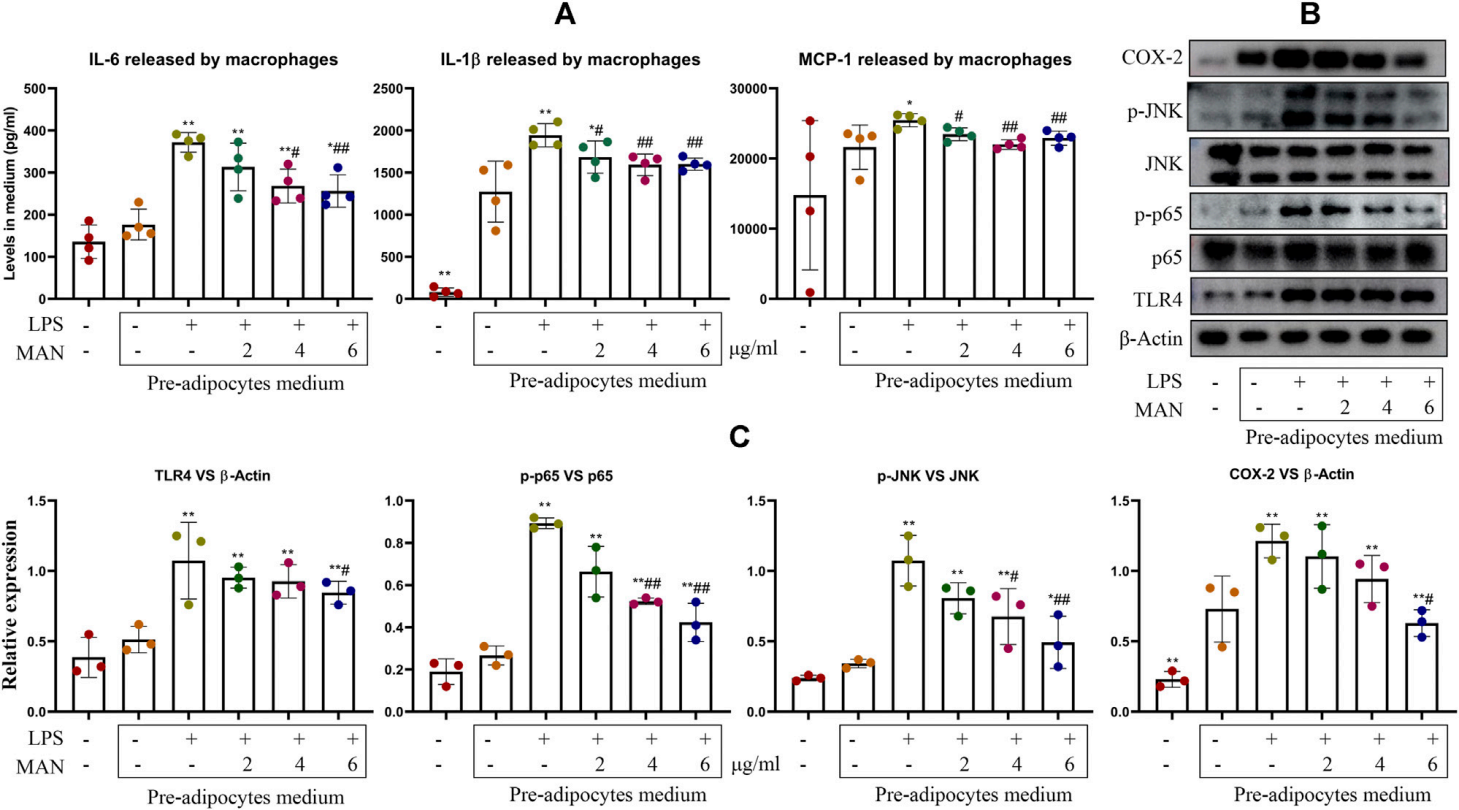
Pre-adipocytes culture medium affected the status of macrophages. **(A)** levels of IL-1β and IL-6 and MCP-1 released by macrophages receiving pre-adipocytes culture medium treatment; **(B)** expression of protein TLR4, p65, p-p65, JNK, p-INK and COX-2 in macrophages obtained from assay **(A)**; **(C)** quantification result of assay **(B)**. Statistical significance: *p < 0.05 and **p < 0.01 compared with macrophages cultured with medium from untreated pre-adipocytes; ^#^ *p* < 0.05 and ^##^ *p* < 0.01 compared with macrophages cultured with medium from LPS-primed pre-adipocytes [*n* = 4 in image **(A)**, *n* = 3 in image **(C)**].

To further test this theory, we treated pre-adipocytes with LPS and MAN beforehand, and used obtained medium to culture macrophages. Interestingly, even the medium from normal pre-adipocytes promoted pro-inflammatory cytokines release. LPS treatment on pre-adipocytes further augmented IL-6, IL-1β and MCP-1 production in macrophages, and the pro-inflammatory potential of pre-adipocytes culture medium were significantly abrogated by MAN (Figure 9A). Expression of COX-2 and TLR4 and phosphorylation of p65 and JNK was regulated in a similar pattern (Figure 9B). Up-regulation of TLR4 and its downstream targets caused by the medium from LPS-primed pre-adipocytes was restored by MAN (Figure 9C). In this experiment, pre-adipocytes and macrophages were cultured separately, and there was no direct contact. Hence, it solidly supported the speculation that pre-adipocytes derived cytokines mediated immune changes of macrophages within WAT.

## Discussion

RA patients tend to develop metabolic disorders, and cardiovascular diseases have been identified as the main RA-related mortality risk (García-Gómez et al., 2014). In early RA, 16-31% patients are diagnosed with metabolic syndrome (Kerekes et al., 2014). As a result, lipid metabolism alteration in RA patients draws intense attention worldwide. It has been uncovered that accumulated lipids have the potentials to perpetuate inflammation in RA patients (Mcgrath and Young, 2015). Besides from pro-inflammatory metabolic intermediates, WAT-released adipokines are believed similarly contributing to RA. Their levels positively correlate to arthritic manifestations (Mcgrath and Young, 2015; Francisco et al., 2019). Therefore, limiting WAT expansion and fat anabolism seem to be beneficial for RA therapies. However, the truth is far more complicated, and several facts doubt this claim. Firstly, RA-related metabolic complication cannot be simply defined as hyperlipidemia. In fact, T-CHO, LDL-C and TG in RA patients are even lower than general population (Mcgrath and Young, 2015). Secondly, although obesity is a risk factor for RA, significant body weight and fat reservoir loss have been reported as arthritis progresses (Chimenti et al., 2015). The body weight loss with the rate >3 kg/m^2^ per year is associated with poor prognosis and high risk of death (Sattar and Mcinnes, 2015). In line with this, well preserved fat reservoir usually promises better prognosis and slighter joints destruction (Stavropoulos-Kalinoglou et al., 2011). Furthermore, the role of adipokine in RA is still need to be further characterized. Leptin was reported to be associated with reduced radiographic joint damage, and adiponectin is usually defined as an anti-inflammatory rather than pro-inflammatory factor (Francisco et al., 2019). Expression of PPAR-*γ*, the key regulator of adipogenesis is deficient in RA patients, and up-regulation of this signaling can sustainably attenuate this disease (Liu et al., 2020). These conflicting evidences eventually lead to the conceptualization of lipid paradox. Until now, this mystery is still un-clarified. Based on results from this study and many other available clues, we attempted to elucidate this puzzle from a global perspective.

Blood glucose was significantly reduced in AIA rats (Table 1). Similar phenomenon was observed by other researchers (Arias de la Rosa et al., 2018). It could be caused by the accelerated aerobic glycolysis, which fuels inflammatory reactions in immune cells (Zhu et al., 2015). MAN restored blood glucose levels in AIA rats, indicating the successful switch of energy supply from NAMPT-mediated glycolysis to SIRT1-mediated oxidative phosphorylation (Liu et al., 2012). This change led to improved energy utilization efficiency, and thereby reduced the reliance on lipid utilization to replenish blood glucose. As a result, fat depletion was eased, and the down-regulated fat oxidation consequently relieved oxidative stress (Figures 3C, 4A). Consistent to this notion, accelerated glycolysis and β-oxidation have been confirmed as a hallmark of RA (Young et al., 2013). We speculated that the abnormal decrease of circulating lipids and fat reservoir in severe RA patients should be caused by increased resting metabolic rate, as fat is utilized as a major alternative energy supply under this situation (Chimenti et al., 2015). From this perspective, restored adipogenesis could serve as an indicator for improved inflammation, which is supported by many available evidences. IL-6 situates in the center of inflammatory cascade. Interestingly, it can substantially suppress lipid anabolism, and decreased TG and T-CHO production was noticed in IL-6-treated mice (Hashizume and Mihara, 2011). The hypolipidemic drugs thiazolidinediones exhibit promising therapeutic effects on RA (Wan et al., 2007). It inevitably brings metabolic consequences, and if accelerated fat anabolism contributes to their anti-rheumatic potential is yet to be clarified. Successful treatment of experimental arthritis is always accompanied with restoration of fat reservoir loss. More importantly, relative high levels of TG, LDL-C and T-CHO are always observed in well-controlled RA patients (Kerekes et al., 2014).

At the meantime, the clinical implication of metabolic alteration should be evaluated dynamically. Restored adipogenesis seems to be favorable for RA therapy during the acute inflammation, but the situation could be totally different at other stages. As well known, obesity provokes inflammation in an attempt to expedite energy expenditure and re-balance energy metabolism (Rocha and Libby, 2009). Consistently, we found culture medium from normal pre-adipocytes promoted IL-1β production in macrophages (Figure 9A). Thus, excessive WAT mass will inevitably increase the odds developing RA by creating an inflammatory environment. Actually, over 30% RA patients exhibit signs of obesity (Stavropoulos-Kalinoglou et al., 2011; Mcgrath and Young, 2015). Lipid-lowering agent like statins can alleviate inflammatory symptoms in RA patients (Soulaidopoulos et al., 2018). That is, adiposity is harmful during the early and pre-clinical stages of RA. Many conflicting observations can be explained by the dynamic lipid metabolism changes. Low circulating lipids are observed during active RA, but the situation in experimental arthritis is largely different. This discrepancy could be attributed to varied disease states. Due to short disease courses, animal arthritis models are more similar to early RA, when hyperlipemia is the major metabolic complication (Kerekes et al., 2014). Central obesity is common in RA patients. But fat loss is deeply involved in the development of rheumatoid cachexia (Soeters and Sobotka, 2012). According to different definition criteria, the incidence of rheumatoid cachexia ranges from 10 to 67%. Interestingly, it is exclusively observed in under- and normal-weight individuals. It prevalence decreases as weight increases, and it barely occurs in obese patients (Stavropoulos-Kalinoglou et al., 2011). Thereby, to improve the nutritional state of RA patients, restoring metabolism homeostasis rather than simply lowering lipids is more rational. The dynamic lipid metabolism reflected in the changes of metabolic regulators too. We found significant changes on levels of NAMPT, SIRT1 and PPAR-*γ* in AIA rats during the peak of secondary inflammation (Figure 2). Comparatively, chronic inflammation during later stage of AIA barely affected them (Figure 4).

Thoroughly investigating excretion profile of WAT is another key to address lipid paradox. Initially, we thought MAM-treated pre-adipocytes eased LPS-induced inflammation in macrophages by reducing pro-inflammatory metabolic by-products. However, the contents of MDA, SOD and GSH in culture medium were too low to be determined using conventional methods, and no decrease in FFAs level was observed. Hence, the most plausible explanation is that these effects were mainly mediated by adipokines, considering the fact that WAT is an important secretion tissue. In line with this, we found that the medium from pre-adipocytes culture imposed significant impact on the production of cytokines in macrophages (Figure 9). Several pro-inflammatory mediators including IL-6 and MCP-1 are defined as adipokines nowadays, and their production are tightly controlled by PPAR-*γ*. Theoretically, activation of PPAR-*γ* can reduce their production due to its suppressive nature on pro-inflammatory pathways (Wahli and Michalik, 2012). Indeed, the secretion of MCP-1, IL-6 and PAI-1 was impaired upon PPAR-*γ* activation in 3T3-L1 adipocytes (Kim and Park, 2015). Here, we specifically highlighted IL-6, as it is required for T cells development. Compared with IL-1β, more profound decrease in levels of IL-6 was achieved upon MAN treatment in AIA rats (Figure 3B). Similar phenomena were also observed *in vitro* (Figures 8A, 9A). Meanwhile, the role of some classic adipocytes in RA is still elusive. As mentioned above, leptin can protect joints from degradation. Although adiponectin is always positively correlated to joint damages, it cannot solidly prove that adiponectin promotes RA progression. In fact, high levels of anti-inflammatory cytokines can be detected in RA joints (Hussein et al., 2008). Many researches have verified the anti-inflammatory nature of adiponectin. Its pathological role in RA was mainly conceived based on *in vitro* experiments, which cannot totally simulate the conditions in RA patients (Francisco et al., 2019). On the other side, some studies even showed that adiponectin benefited the improvement of RA-related inflammation (Senolt et al., 2006). The abnormal increase of adiponectin therefore could be part of immune re-balancing mechanism in the response to inflammatory stimulus. Collectively, we cannot simply judge if WAT excretion is good or bad for RA, and we should emphasize more on immune phenotypes of (pre)-adipocytes rather than WAT mass in further researches.

It was extensively observed that MAN treatments intervened in glucose and lipid metabolism (Chen et al., 2018). Apart from its anti-diabetic potentials, effects of MAN on lipid metabolism are especially attractive. Most researchers believe MAN hampers adipocytes differentiation (Quan et al., 2012; Nelli et al., 2013; Taher et al., 2015). However, the conclusion is doubtful. Quan et al. (2012) believed that anti-obesity effect of MAN was mainly attributed to its negative effects on adipocytes viability. Unfortunately, our previous investigation suggested that the concentrations adopted in their study cannot be achieved *in vivo* at normal oral doses (Xu et al., 2017). Besides, intense MAN stimulus will cause nonselective cytotoxicity due to the breakdown of mitochondria integrity (Martínez-Abundis et al., 2010). Under this circumstance, the obtained result is quite misleading and unreliable. Similarly, Taher et al. (2015) claimed that MAN impaired adipocytes differentiation through down-regulation of PPAR-*γ* using the concentration up to 25 μM. Other reports revealed that MAN promoted PPAR-*γ* expression in adipocytes under inflammatory conditions (Bumrungpert et al., 2010; Choi et al., 2015). PPAR-*γ* is identified as the most powerful regulator driving adipogenesis (Wahli and Michalik, 2012). Thereby, the exact effect of MAN on fat metabolism is still to be further investigated. At least, the current study suggested that MAN could have diversified effects under different pathological conditions. It suppressed fat synthesis in obese subjects, but restored fat reservoir in AIA rats. The varied immune circumstances could be decisive. Even in this study, we found MAN exerted totally opposite effects on SCD-1 under different situations.

## Conclusion

The current study confirms that the anti-rheumatic potentials of MAN are related to its metabolism regulatory properties. MAN up-regulated PPAR-*γ* in (pre)-adipocytes. As a result, it boosted fat anabolism, and consequently impeded glycolysis-driven inflammatory polarization of monocytes/macrophages in AIA rats. The in vitro experiments confirmed these findings, and further demonstrated that the reshaped secretion profile of adipocytes also greatly contributed to the eased inflammation controlled by monocytes/macrophages.

## Data Availability

The raw data supporting the conclusion of this article will be made available by the authors, without undue reservation.
