# Correction: α-Mangostin alleviated inflammation in rats with adjuvant-induced arthritis by disrupting adipocytes-mediated metabolism-immune feedback

**DOI:** 10.3389/fphar.2025.1671368

**Published:** 2025-08-21

**Authors:** Ying-Hao Hu, Jun Han, Lin Wang, Chao Shi, Yan Li, Opeyemi Joshua Olatunji, Xiu Wang, Jian Zuo

**Affiliations:** ^1^ Department of Traditional Chinese Medicine, The First Affiliated Hospital of Wannan Medical College (Yijishan Hospital), Wuhu, China; ^2^ Research Center of Integration of Traditional Chinese and Western Medicine, Wannan Medical College, Wuhu, China; ^3^ Drug Research and Development Center, School of Pharmacy, Wannan Medical College, Wuhu, China; ^4^ Department of Pharmacy, The First Affiliated Hospital of Wannan Medical College (Yijishan Hospital), Wuhu, China; ^5^ Faculty of Traditional Thai Medicine, Prince of Songkla University, Hat Yai, Thailand; ^6^ Key Laboratory of Non-coding RNA Transformation Research of Anhui Higher Education Institution, Wannan Medical College, Wuhu, China

**Keywords:** macrophage, rheumatoid arthritis, PPAR-γ, adjuvant-induced arthritis, fat metabolism, adipocytes

There was a mistake in Figure 3C as published. A tissue image from a concurrently conducted study was erroneously used. The corrected Figure 3 appears below.

**FIGURE 3:**
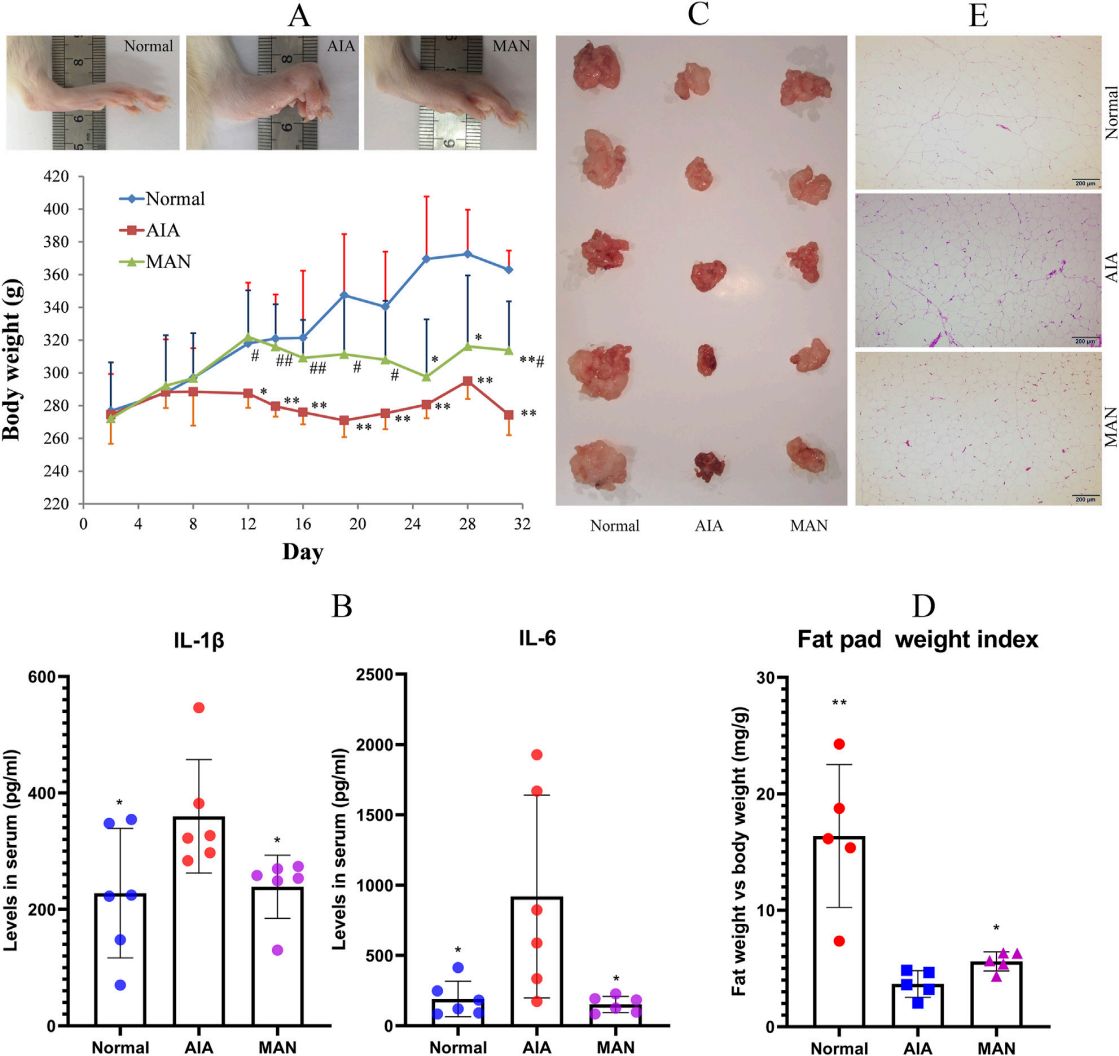
MAN restored fat reservoir loss in AIA rats. **(A)** local inflammation in paws and body weight changes of rats; **(B)** levels of IL-1β and IL-6 in rat serum; **(C)**, images of perirenal fat pad; **(D)** relative weight index of fat pad; **(E)** histological examination of WAT. Statistical significance: *p < 0.05 and **p < 0.01 compared with AIA rats [*n* = 6 in image **(A,B)**, *n* = 5 in image **(D)**].

The original article has been updated.

