# Supplementary figures and images for: α-Mangostin Alleviated Inflammation in Rats With Adjuvant-Induced Arthritis by Disrupting Adipocytes-Mediated Metabolism-Immune Feedback
Source: Front Pharmacol. 2021 Jul 7;12:692806. doi: 10.3389/fphar.2021.692806 (PMC8293671; doi:10.3389/fphar.2021.692806)

Figure 4

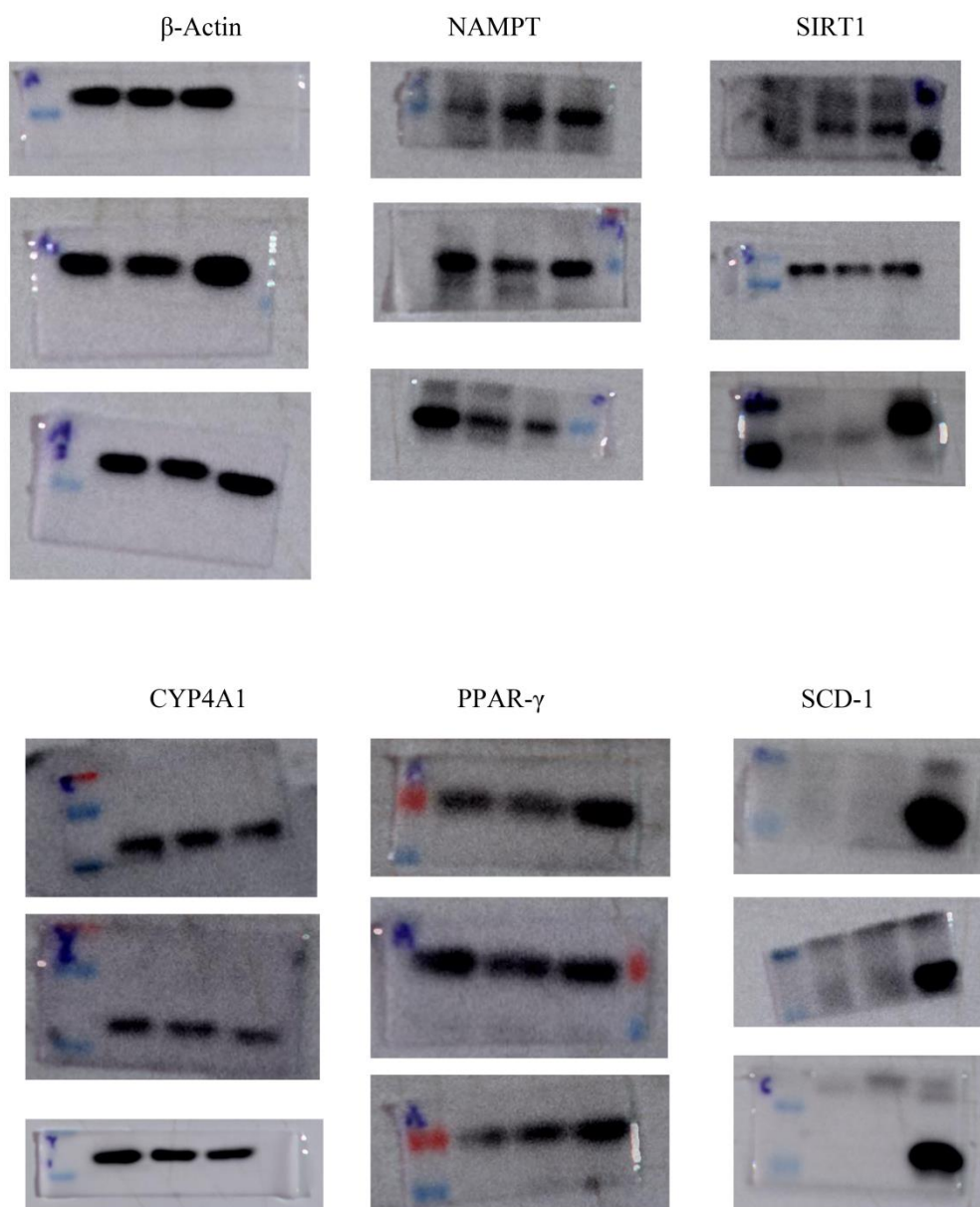

Figure 6

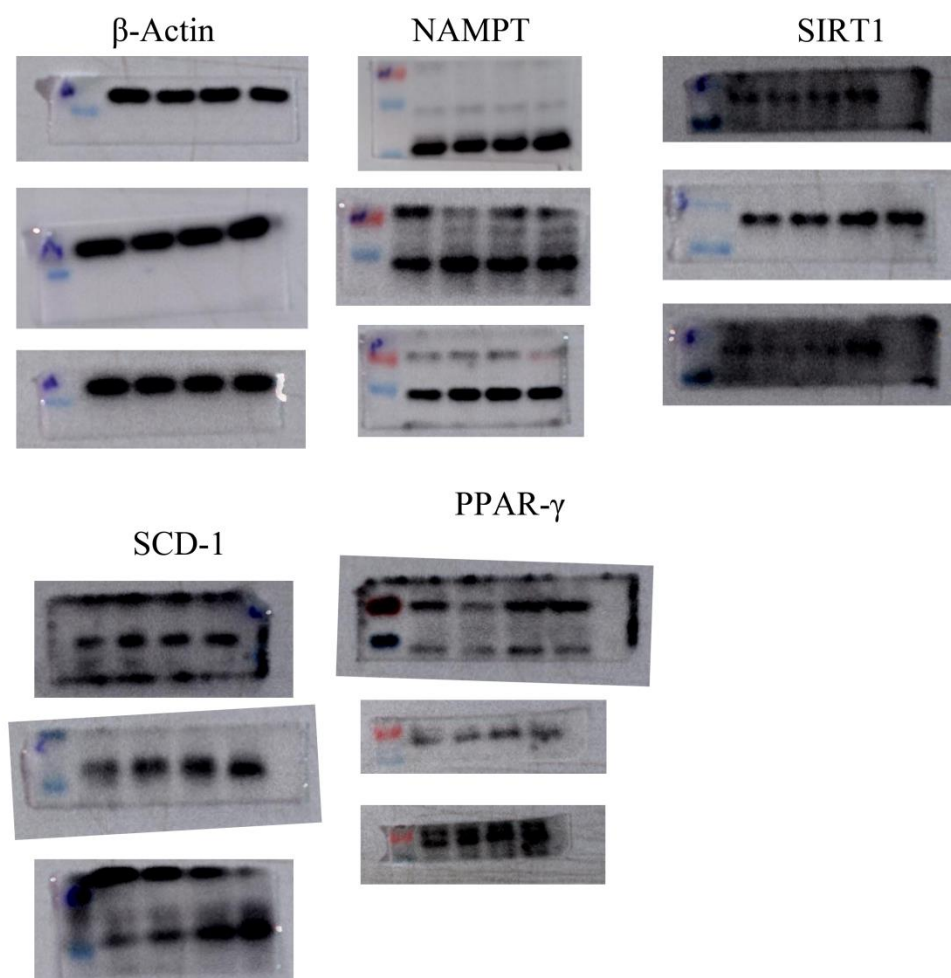

Figure 7

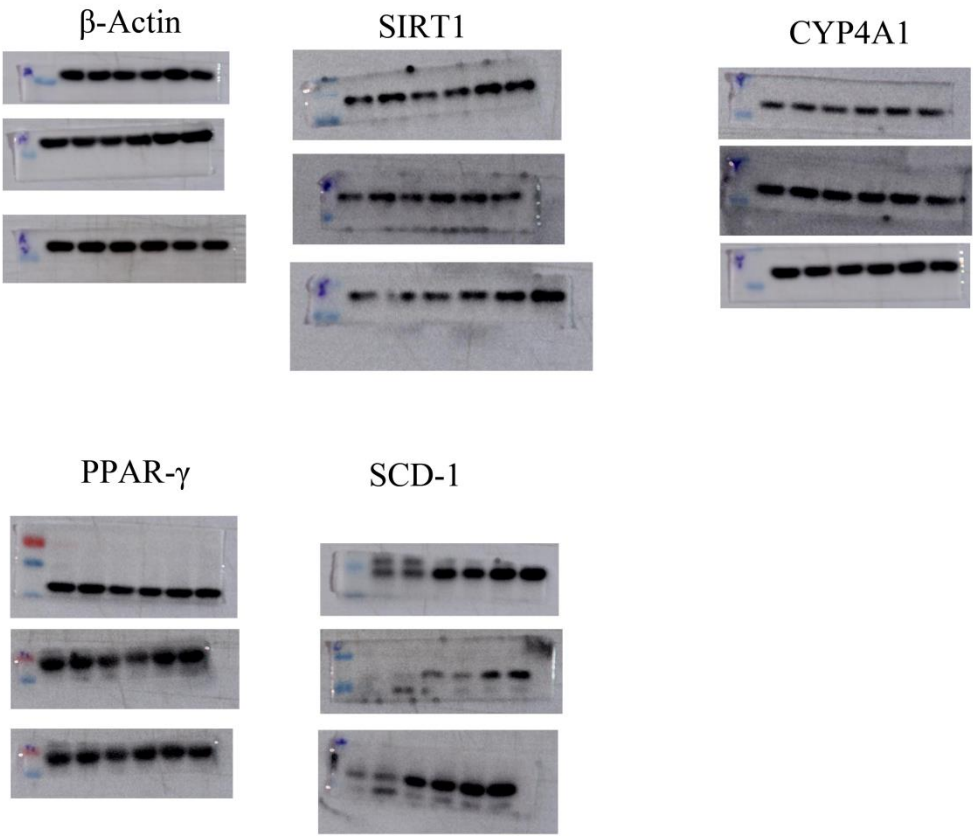

Figure 8

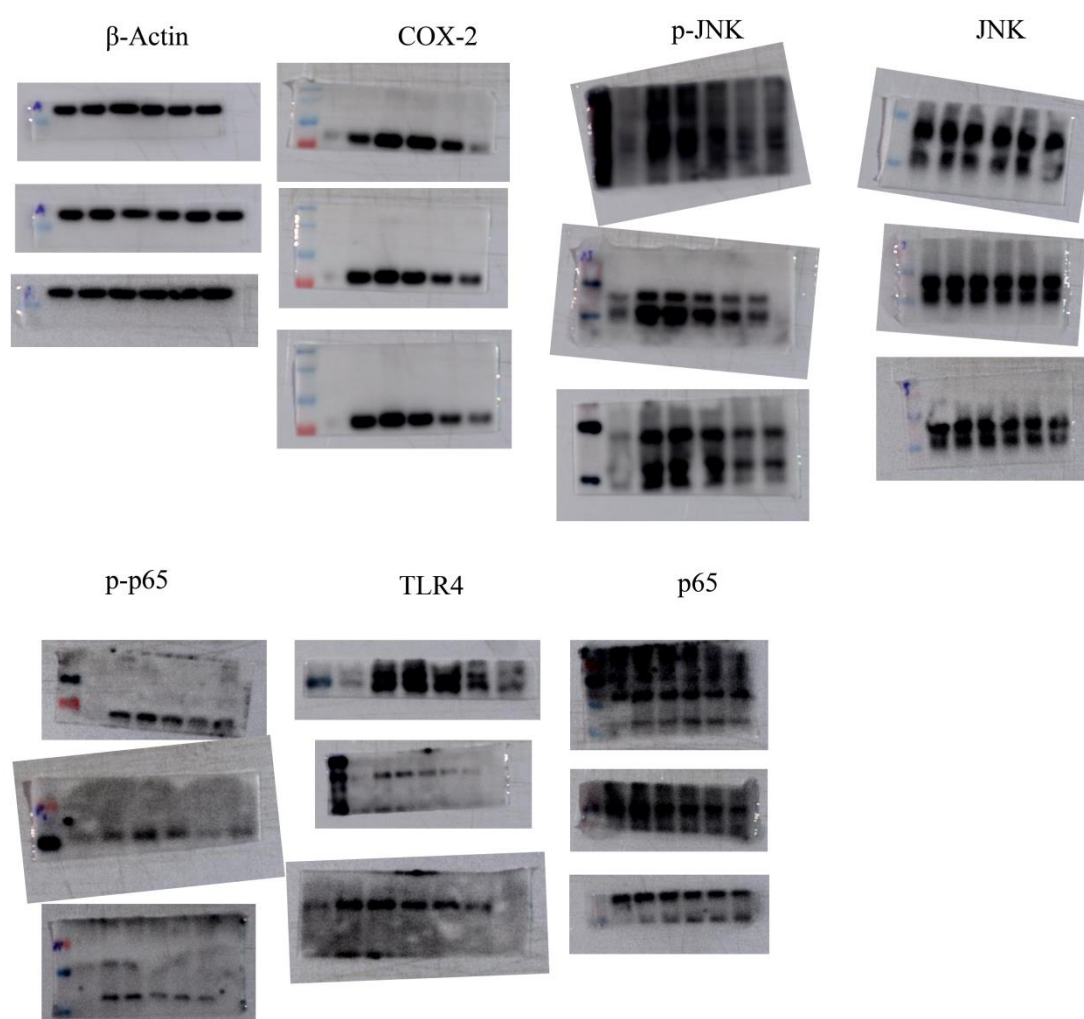

Figure 9

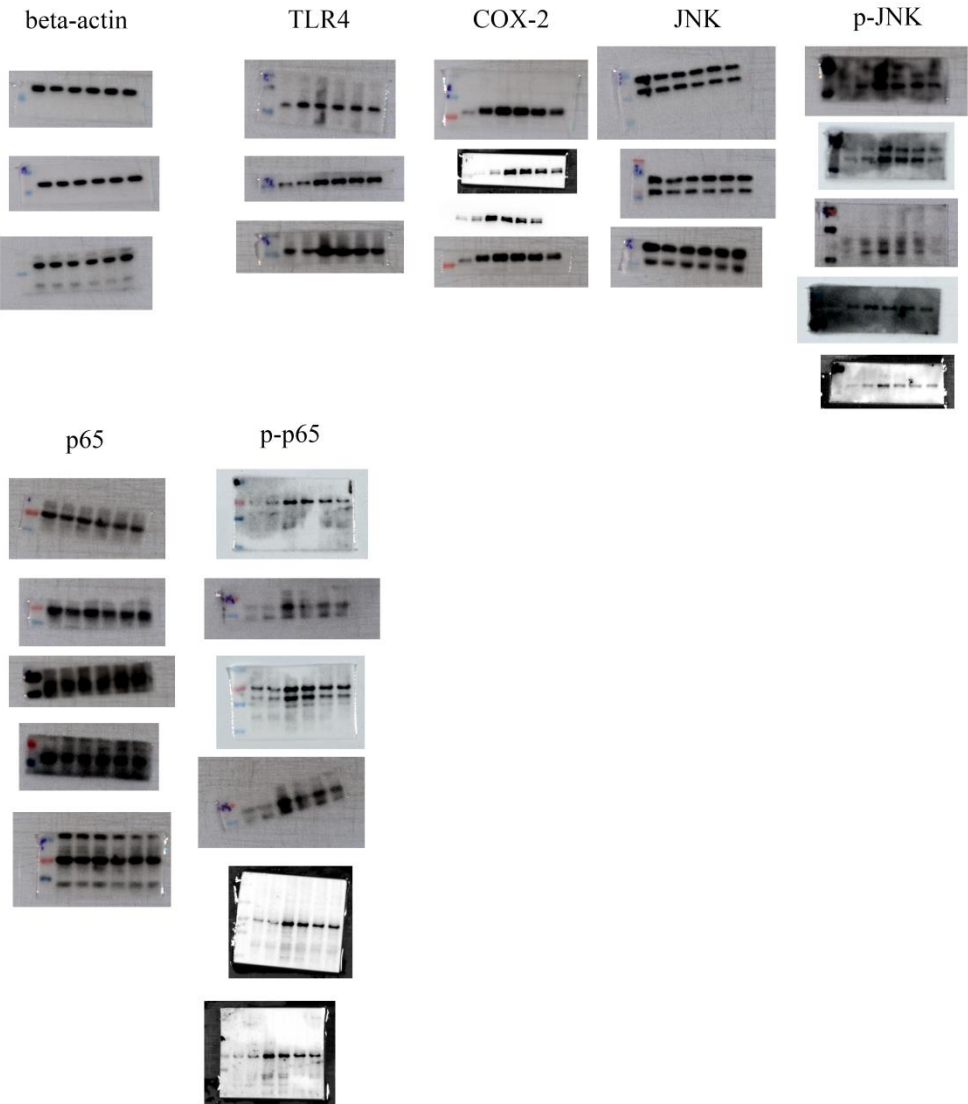

Supplement: Supplementary file 1 [file DataSheet2.PDF]
