# Supplementary material for: α-Mangostin Alleviated Inflammation in Rats With Adjuvant-Induced Arthritis by Disrupting Adipocytes-Mediated Metabolism-Immune Feedback
Source: Front Pharmacol. 2021 Jul 7;12:692806. doi: 10.3389/fphar.2021.692806 (PMC8293671; doi:10.3389/fphar.2021.692806)
Supplement: Supplementary file 2 [file DataSheet1.PDF]

Premier sequences

Gene	Forward	Reverse
β-Actin	5′-TGTCCACCTTCCAGCAGATGT-3′	5′- AGCTCAGTAACAGTCCGCCTAGA-3′
SIRT1	5′- GTGTCATAGGTTAGGTGGCGAG-3′	5′- GAAGAGGTGTTGGTGGCAACTC-3′
NAMPT	5′- GATTCTGGAAATCCGCTCGA-3′	5′- TGACTCTAAGGTAAGGTGGCAGC-3′
PPARγ	5′-GGAGATCCTCCTGTTGACCC-3′	5′-TGGGTCAGCTCTTGTGAACG-3′
SCD-1	5′-TCGTCAGCACCTTCTTGAGATA -3′	5′-GTTGATGTGCCAGCGGTACT-3′
IL-1β	5′-TCCTCTGTGACTCGTGGGAT-3′	5′- TCAGACAGCACGAGGCATTT-3′
iNOS	5′-TGCCTTTGCTCATGACATCG-3′	5′-AACACGTTCTTGGCGTGGA-3′
